# Mechanistic insights into protein folding by the eukaryotic chaperonin complex CCT

**DOI:** 10.1042/BST20220591

**Published:** 2022-10-05

**Authors:** Theresa M. Smith, Barry M. Willardson

**Affiliations:** Department of Chemistry and Biochemistry, Brigham Young University, Provo, UT 84602, U.S.A.

**Keywords:** cryo-electron microscopy, molecular chaperones, molecular mechanisms, protein conformation

## Abstract

The cytosolic chaperonin CCT is indispensable to eukaryotic life, folding the cytoskeletal proteins actin and tubulin along with an estimated 10% of the remaining proteome. However, it also participates in human diseases such as cancer and viral infections, rendering it valuable as a potential therapeutic target. CCT consists of two stacked rings, each comprised of eight homologous but distinct subunits, that assists the folding of a remarkable substrate clientele that exhibits both broad diversity and specificity. Much of the work in recent years has been aimed at understanding the mechanisms of CCT substrate recognition and folding. These studies have revealed new binding sites and mechanisms by which CCT uses its distinctive subunit arrangement to fold structurally unrelated substrates. Here, we review recent structural insights into CCT-substrate interactions and place them into the broader context of CCT function and its implications for human health.

## Introduction

All organisms depend on properly folded and functioning proteins for health and survival. Although often taken for granted, protein folding is a complex process; a linear, highly flexible polypeptide chain populated by side chains with a wide variety of chemical properties must collapse on itself to find the specific conformation which renders it both energetically stable and able to perform its biological function. While most small proteins fold rapidly on their own, many larger proteins with complex topologies are prone to misfolding — becoming trapped in alternate states by an insurmountable free energy barrier. At best, a misfolded protein is rendered non-functional. At worst, exposed hydrophobic patches cause the misfolded protein to aggregate and trigger the misfolding of neighboring proteins, resulting in a highly toxic chain reaction of amyloid fiber formation. Indeed, several diseases including Parkinson's and Alzheimer's diseases involve a breakdown of proper protein folding and the buildup of amyloid fibers [1]. Survival therefore depends on molecular chaperones, a class of proteins that stabilize and promote the proper folding of other proteins. Chaperones act by at least one of three functions: holding unstable subunits until they can find binding partners, folding nascent peptide chains, and disassembling aggregates. They tend to be quite promiscuous, serving a wide variety of client proteins by recognizing exposed hydrophobic residues. There are several unrelated major classes of chaperones that promote nascent protein folding in an ATP-dependent manner. These include the Hsp70 and Hsp90 families as well as the Hsp60 chaperonin family [2].

## The chaperonins

**Figure 1.**
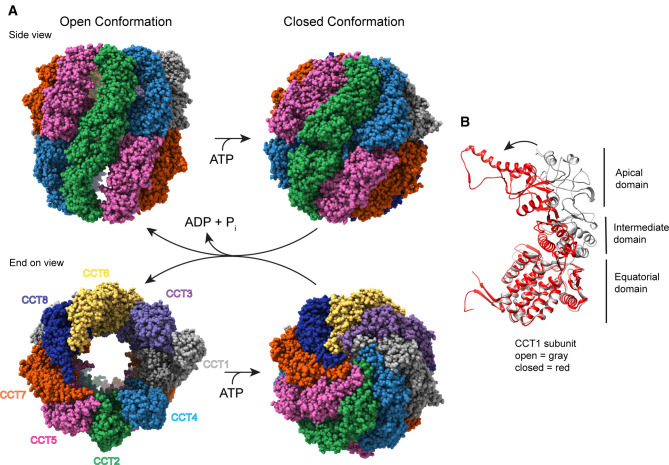
Structure of CCT. (**A**) Side and end-on views of human CCT with and without ATP (adapted from PDB 7NVN [32] and 6QB8 [12], respectively), highlighting the double ring arrangement of the eight subunits and the conformational change that occurs in the ATP hydrolysis transition state to cap the central folding chambers of CCT. (**B**) A ribbon view of the CCT1 subunit showing the domain structure common to all the subunits and the conformational change between the nucleotide-free (gray) and ATP hydrolysis transition state (red) that closes the CCT folding chamber.

Chaperonins are large complexes ∼1 MDa in size defined by a structure comprised of two stacked rings of 7–9 subunits with a protein folding chamber in the center of each ring. These subunits may be identical or homologous depending on the species. Each subunit consists of three domains: the apical domain, which recognizes and binds substrates, the equatorial domain, which binds ATP and forms the inter-ring contacts, and an intermediate domain, which conveys conformational information between the other two (Figure 1). Chaperonin substrates are encapsulated and folded within the large central chambers in an ATP-dependent manner (Figure 1) [3,4]. Group I chaperonins are found in prokaryotes and organelles of prokaryotic origin. Their rings consist of 7 identical subunits and require an Hsp10 co-chaperone to act as a lid to close the folding chamber. The extensively studied GroEL/ES system in bacteria belongs to this group [4]. Group II chaperonins are found in archaea and eukaryotes. In contrast with Group I, their rings are made of 8 or 9 subunits that don't require an additional co-factor. Instead, helical extensions in their apical domains fold inward to close the chamber upon ATP hydrolysis and form a built-in lid (Figure 1B) [5].

## CCT/TRiC

The cytosolic chaperonin containing TCP-1 (CCT, also known as TRiC) is the Group II chaperonin in eukaryotic cells. The most complex of all the chaperonins, its rings are composed of eight distinct subunits in a specific arrangement (Figure 1) [6]. This subunit arrangement arose early in the evolution of eukaryotic cells and is universally conserved throughout the domain, allowing for a dramatic increase in diversity and specificity in substrate interactions over bacterial or archaeal chaperonins that have just one or two isoforms in their ring structures [7,8]. Indeed, the emergence of CCT was likely a key factor in the rise of eukaryotic proteome complexity as CCT has been reported to interact with an estimated 10% of the cytosolic proteome [9,10]. Proteins folded by CCT are typically referred to as substrates, and many of these substrates cannot be folded by any other chaperone. Consequently, CCT function is essential for the survival of eukaryotic cells. The most abundant substrates are the cytoskeletal proteins actin and tubulin, which must be folded by CCT prior to forming microfilament and microtubule structures [11]. In addition to protein folding, CCT contributes to the assembly of protein complexes by first folding one or more subunits of the client complex and then providing a platform on which the complex can assemble or by releasing the subunit as it associates with its binding partners [12–15]. There is also evidence that CCT assists in protein quality control by interacting with distressed or aggregated proteins, particularly those containing aggregation-prone poly-Q tracks such as Huntingtin, and sequestering them until they can be degraded [16,17].

The mechanism of CCT substrate recognition and targeted folding has been the subject of debate for some time. Its substrates vary widely both in topology and size with little in common besides a tendency towards complex, multi-domain folds or WD-40 repeat domains that form β-propeller structures [10,12,13,18,19]. However, CCT is not nearly as promiscuous as other chaperones such as the Hsp70s which simply bind to exposed hydrophobic patches [20], indicating that there must be a mechanism of specificity. The original theory of CCT function was the Anfinsen cage model which postulated that substrates initially bound to the apical domains of open CCT and then became encapsulated in one of the two folding chambers upon ATP hydrolysis. Folding would then be facilitated simply by constraints placed on flexibility by the walls of the chamber and by sequestration away from other unfolded peptides that could cause aberrant binding [21]. This mechanism is similar to how GroEL folds its substrates and may well be true of some CCT substrates [4]. However, recent work paints a much more complex picture of CCT function, demonstrating the capability of an active and substrate-specific role in polypeptide folding. Here we will focus on this work and other recent developments in our understanding of CCT function.

## ATP binding and asymmetry

**Figure 2.**
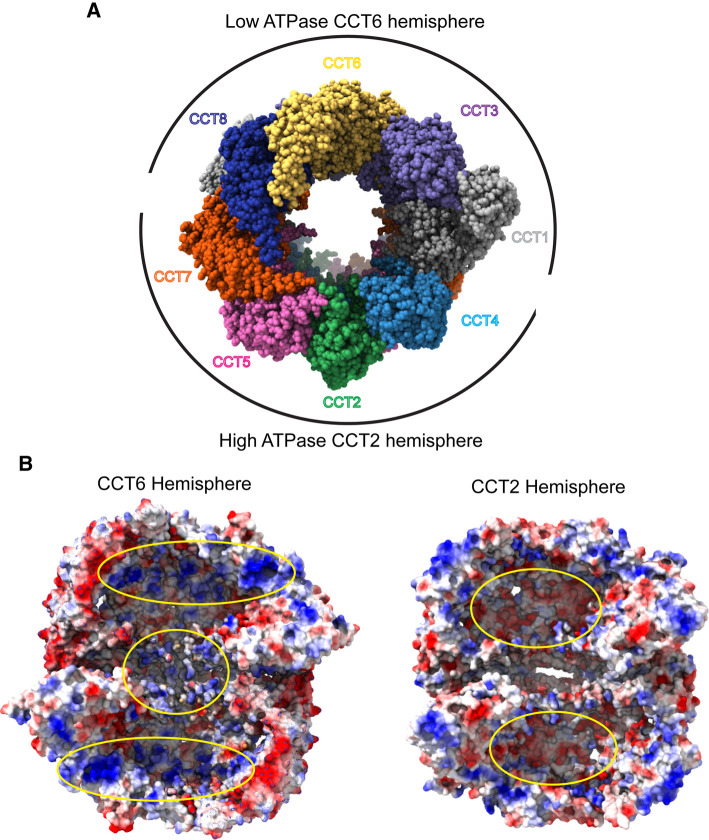
Functional differences between the CCT hemispheres. (**A**) Open form of human CCT (PDB 6QB8) showing the hemispheres centered around CCT2 and CCT6 with high ATPase activity in the CCT2 hemisphere and low ATPase activity in the CCT6 hemisphere. (**B**) Electrostatic surface views of the interior of the closed form of human CCT (PDB 7NVN) with the CCT6 hemisphere on the left and the CCT2 hemisphere on the right. (PDB 7NVN, positive charge = blue, neutral = white, and negative charge = red.) For the CCT6 hemisphere, the CCT2, 4, 5 and 7 subunits have been removed to reveal the interior of the folding chambers. Positively charged patches in the upper and lower chambers and between the CCT rings are highlighted by yellow ovals. For the CCT2 hemisphere, the CCT1, 3, 6 and 8 subunits have been removed to view the interior of the folding chambers. Negatively charged patches in the upper and lower chambers are highlighted by yellow ovals.

The helical extensions of group II chaperonins spiral inward to close the folding chamber in ATP-dependent manner (Figure 1). This built-in lid begins to close as subunits bind ATP, and it completes the process during the transition state of ATP hydrolysis, which has been demonstrated by the widespread use of ADP-AlF_x_ to mimic ATP hydrolysis and trap CCT in a closed conformation for structural studies [22]. After the phosphate is released, the now ADP-bound chaperonin reverts to the open state until the ADP is once again exchanged for ATP. While ATP hydrolysis is a cooperative process, demonstrating positive intra-ring and negative inter-ring cooperativity [23,24], ATP binding has been shown to be a stochastic process for archaeal chaperonins, consistent with the high degree of conservation of their ATP binding pockets [25]. This conservation is retained in CCT, however CCT is unique in that its subunits do not bind or hydrolyze ATP at equal rates. Instead, CCT is divided into two hemispheres; a high ATPase CCT2 hemisphere comprising CCT 2, 4, 5, and 7, that drives ring closure, and a low ATPase CCT6 hemisphere, comprising CCT 1, 3, 6, 8 [26–29] (Figure 2). CCT 6 and 8 hardly bind ATP at physiological concentrations [26,28], and mutagenesis of their active sites has little to no effect on CCT activity or cell viability in yeast [26,30], suggesting that they do not contribute to ATP hydrolysis. The effect of this one-sided ATP hydrolysis is an asymmetric ‘power stroke' in which CCT closes its folding chamber sequentially in a defined order [26–29,31]. This power stroke may have a tendency to push substrates towards the low-affinity side over the course of folding as the majority of substrates for which structural analysis is available seem to form more contacts with CCT 3, 6, and 8 [12,13,32,33].

The reason for the difference in ATP binding affinities among the CCT subunits stems from variability in the nucleoside binding residues. Unlike the triphosphate-contacting residues, which are conserved through all subunits, those that contact the nucleoside are fairly conserved in the high-affinity subunits, but less so in the low-affinity subunits. Cryo-EM structures of open human [12] and yeast CCT [27] showed the nucleotide binding pockets of the subunits were empty except for CCT 6 and CCT 8, which were still bound to ADP. Analysis of the interactions between ADP and the binding pocket identified several residues with potential roles in stabilizing the ADP bound state, including K171 in CCT8 which forms an extra salt bridge with the nucleoside. CCT 6 and 8 also both have an aspartate at position 499 which appears to be close enough to hydrogen bond with the ribose ring, while other subunits have either glutamate or glutamine at the same position which would sterically displace ADP [12]. Thus, lack of ATP hydrolysis by the low-affinity hemisphere is likely driven by residues in the nucleotide binding pocket that stabilize bound ADP and prevent nucleotide exchange rather than disrupted ATP hydrolysis.

Substrate folding trajectories are also mediated by asymmetry of charge distribution within the CCT chamber itself. High resolution structures of closed CCT bound to β-tubulin showed that upon closure, a large patch of positively charged residues is exposed across the CCT6 hemisphere while a negative patch is found across the CCT2 hemisphere (Figure 2). Negatively charged surfaces of β-tubulin interacted with the positively charged patch on the CCT6 hemisphere to stabilize intermediates in β-tubulin folding [33], confirming predictions of electrostatic interactions between CCT and substrates from earlier studies [34,35]. Thus, asymmetric substrate binding and ATP hydrolysis increase the complexity of CCT and add to the tools available to help substrates fold.

## Substrate recognition and binding

CCT folds a wide range of specific, yet diverse substrates ranging from actin and tubulin, to the β subunits of G protein heterotrimers (Gβ) and the 150 kDa RAPTOR subunit of the mammalian target of rapamycin complex 1 (mTORC1) [12,13,36]. Although it is generally accepted that the apical domains play an important role in recognition of substrates, the precise interactions and sequence elements for CCT are not yet fully understood. Joachimiak et al. [37] partially addressed this question by mapping the binding sites of well-characterized CCT substrates to the apical domains of their cognate CCT subunits. The results identified a shallow groove in the apical domain between the proximal loop and helix 11 lined with combinations of polar and non-polar residues unique to each subunit. The non-polar residues contributed to the binding affinity as is common among chaperones, which generally bind their substrates via exposed hydrophobic patches. The polar residues contributed to specificity and helped to properly orient the subunit [37]. This model explains the range of specificity and the lack of common motifs among CCT substrates because the variations among the subunits allow a great deal of flexibility in the chemical nature of substrate interactions. Substrates can bind to different subunits in specific orientations optimized to their individual folding trajectories. Furthermore, this study proposed a mechanism by which substrates would be dislodged from their binding site on one subunit by steric interference from a loop from an adjacent subunit upon ring closure [37]. The asymmetric nature of CCT ring closure would provide an extra layer of complexity by dictating the order of substrate release into the folding chamber.

A more recent study involving cryo-EM and hydrogen-deuterium exchange analysis of denatured actin added to purified CCT in different nucleotide binding states corroborated this model, showing unfolded actin spread out among the apical domains of nucleotide-free CCT. The actin appeared to partially release upon ATP binding in a sequential manner [29]. In contrast, a string of recent cryo-EM structures, which depict various substrates bound to CCT in the open nucleotide-free state, have revealed substrate density situated between the two rings of CCT, associating with the equatorial domains and the N -and C- termini of the CCT subunits rather than bound up in the apical domains (Figure 4a) [12,32,33]. These observations suggest that substrate recognition and folding is more complex and that multiple regions of CCT play a role.

## Structural insight into the folding mechanism

**Figure 3.**
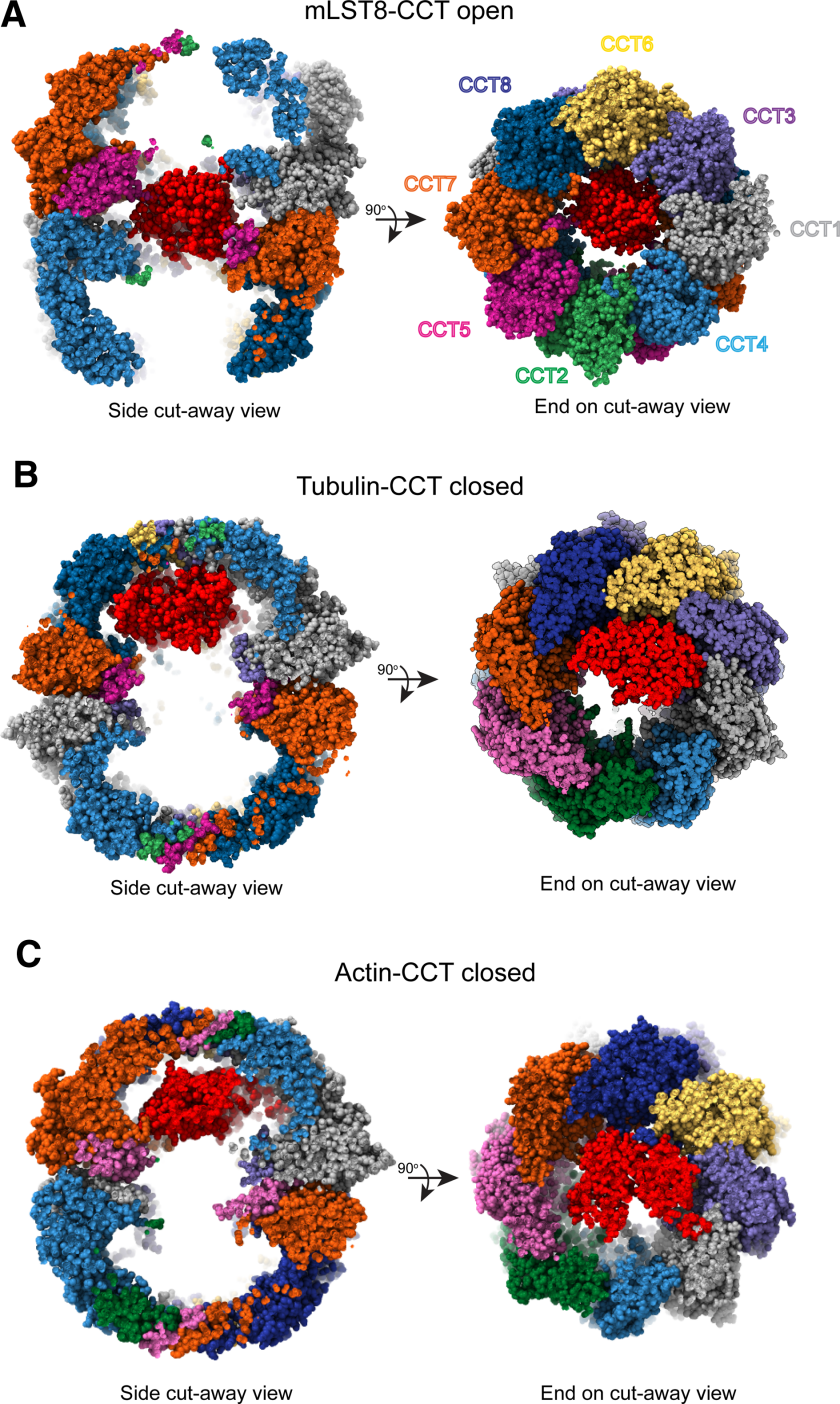
Substrate binding positions within CCT. (**A**) Side and end-on cut away views of mLST8 (PDB 4JT6) sitting between the rings in the open form of human CCT (PDB 6QB8) [12]. (**B**) Side and end-on cut away views of β-tubulin in the folding cavity of closed CCT (PDB 7NVN) [32], highlighting the position of tubulin bound in the apical domains of the CCT6 hemisphere. (**C**) Side and end-on cut away views of actin in the folding cavity of closed CCT (PDB 7NVM) [32], showing actin spanning the cavity to interact with both hemispheres. Substrates are shown in red.

Many CCT substrates to date have been biochemically characterized, however molecular details on the folding mechanism are lacking. Recent high-resolution cryo-EM structures of actin and tubulin as well as the β-propeller protein mLST8 of the mTOR complex have revealed important aspects of the folding mechanism (Figure 3). Here we present a summary of the major insights from these studies.

### Substrate binding between the rings

**Figure 4.**
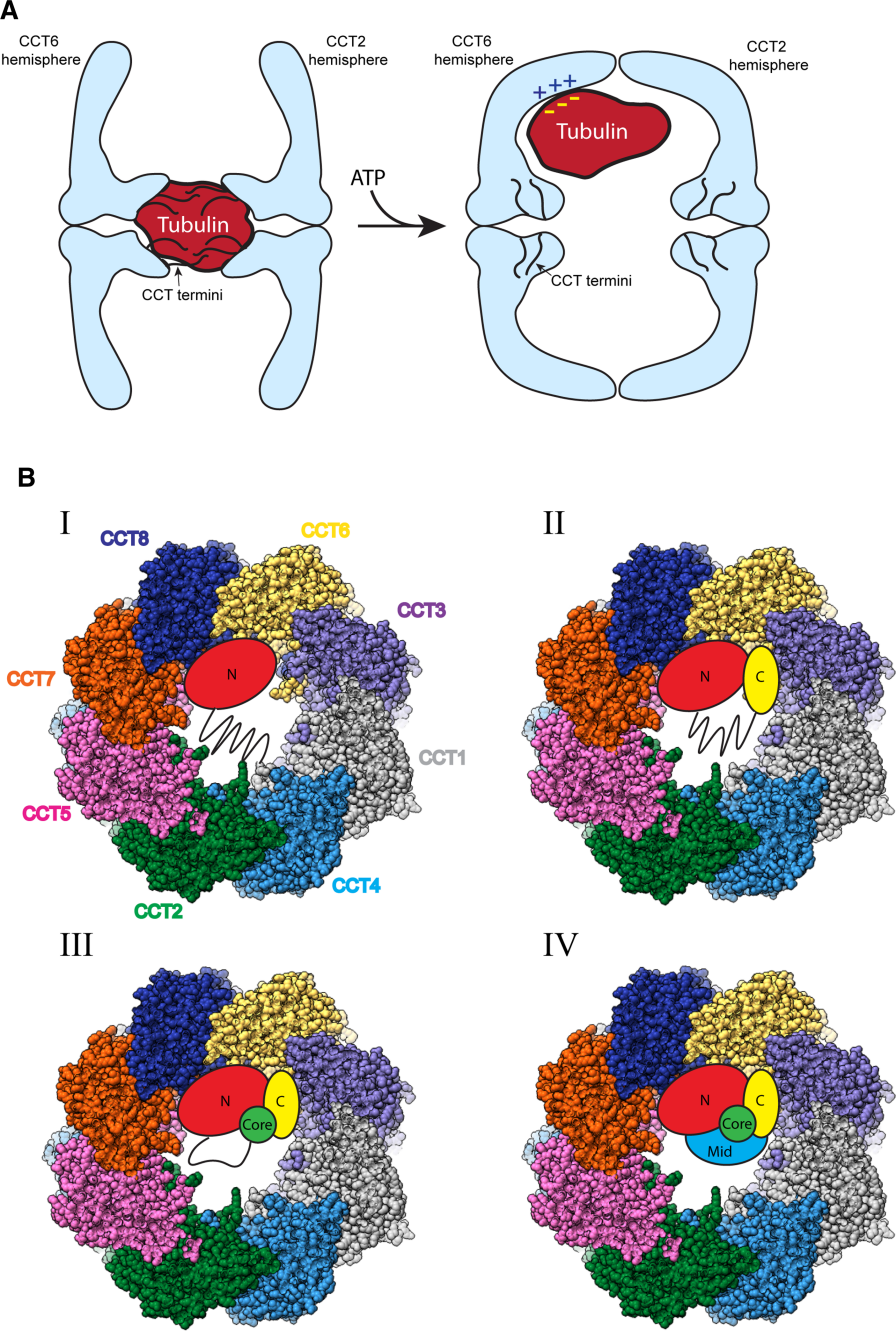
Folding trajectory of tubulin. (**A**) Model showing the position of tubulin within the open (left) and closed (right) conformations of CCT. In the open form, tubulin is in an amorphous state interacting with the N- and C-termini of the CCT subunits (black lines). In the closed form, the tubulin is released from between the CCT rings as the termini redistribute. The tubulin moves into the CCT folding chamber and interacts with positively charged patches in the apical domains along the inner walls of the chamber. (**B**) Model representing the four observed states of tubulin folding inside the folding cavity of closed CCT (PDB 7NVN). The domains of tubulin are indicated: N = the N terminal domain, C = the C terminal domain, Core = the helical core domain, Mid = the middle domain. Lines represent unstructured regions in each folding state. See text for details.

CCT has been observed to bind substrates in the apical domains in its open state and release them into its central chamber in the closed state [29]. The possibility of other substrate binding sites on CCT, beyond the apical domains has been recently raised. The N- and C- termini of CCT, which extend as disordered polypeptide segments into the space between the two rings, were thought to create a barrier between the folding chambers and not otherwise contribute to substrate folding [38,39]. However, this idea was challenged when the cryo-EM structure of mLST8 bound to human CCT in the open state showed a weakly resolved mass corresponding to the size of mLST8 sitting between the rings [12] (Figure 3A). This mass was attributable to mLST8 because it was not observed in the structure of human substrate-free CCT [12]. Another structure has since been reported of tubulin-bound CCT in the open state [33]. This structure also showed a dense, but low-resolution mass, attributable to tubulin, sitting between the rings and interacting with the termini, while the closed form showed well-defined tubulin inside the folding chamber itself [33]. This observation suggests an additional step in the folding of some substrates in which the substrate, possibly after initially binding to the apical domains, is transferred to the CCT termini (Figure 4A). Given the low resolution of these substrates, it appears that the termini hold them in a metastable state to protect them from folding into non-native structures. These interactions would keep the substrate in a compact form, while still maintaining flexibility. Notably, in the closed form the CCT termini pack against the equatorial domains and are much better resolved than in the open form [32], suggesting that a change in position of the termini upon CCT closure releases the substrate from between the rings into the folding chamber where it interacts with newly exposed residues on the chamber walls (Figure 4A) [33]. Consistent which this notion, currently all structures of CCT-bound substrates in the closed form show the substrate in the folding chamber, interacting predominantly with the apical domains, and not between the rings (Figure 3B,C) [29,32,33].

### Directed folding

GroEL and other chaperonins appear to fold their substrates via a mechanism in which substrate folding is mediated by restricted degrees of freedom and charge repulsion upon encapsulation [4]. Growing evidence demonstrates that substrate folding by CCT is a much more complex process with the chaperonin actively directing the folding pathway. Both actin and tubulin have now been shown to make specific contacts with CCT upon binding inside the closed chamber, which make use of the inherent asymmetry in the closed ring and available binding surfaces to ensure their domains are folded in the proper order. Tubulin was contacting CCT 6, 8, and 1 and actin was extended with one domain binding CCT 3 and 6 and another binding CCT 2, 7, and 8 (Figure 3B,C) [32]. For both proteins, the residues involved in the interface with CCT were highly conserved, suggesting that their respective binding interactions are essential for proper folding. The fact that actin and tubulin bind to different sites is significant because it points to different CCT folding mechanisms for different proteins rather than a single universal folding mechanism.

A very recent, in-depth cryo-EM analysis of tubulin bound to CCT has uncovered important details of the mechanism of CCT-dependent protein folding [33]. In the open form, tubulin was located between the CCT rings in an amorphous state, interacting with the N- and C-termini of the CCT subunits. In the closed form, tubulin redistributed into one folding chamber, interacting extensively with positively charged residues in the apical domains on the CCT6 hemisphere. (Figure 4A). 3D classification of the tubulin cryo-EM density revealed four distinct folding states (Figure 4B). One state showed tubulin in an extended conformation with only its N-terminal domain folded and bound to the apical domains of CCT 6 and 8, while its C-terminal hook was bound to a pocket between CCT 1 and 4, bringing the N-terminal domain into proximity with the C-terminus. All these contacts involved electrostatic interactions between positively charged regions of CCT and negatively charged regions of tubulin. In the second state, the folded regions of tubulin and contacts with CCT from the first state were preserved, but the C-terminal domain was also folded and made additional electrostatic interactions with CCT1 and CCT3. The central region of the protein extended into the folding chamber in an unfolded state while making contacts with the C-terminal tail of CCT2. In the third state, more of the central region was folded and all the original contacts with CCT were maintained. In the fourth state, tubulin was fully folded and still bound to CCT. Between states 1–4, the central β-sheet of tubulin was progressively folded, suggesting a folding trajectory for tubulin mediated by interactions with CCT [33].

## CCT co-chaperones

Several co-chaperones of CCT have been identified that modulate its function and play important roles in folding certain substrates. These include prefoldin and the phosducin-like proteins (PhLPs). There are four mammalian PhLPs known to be involved with CCT [40]. Of these, the role of PhLP1 is the best characterized in folding of the β-propeller domains of G protein β subunits. PhLP1 forms a ternary complex with CCT and Gβ1 that is essential for Gβ1 folding. Structural studies show that PhLP1 binds to the apical domains of CCT above Gβ1, which sits lower in the CCT folding chamber [13]. Interactions of PhLP1 with Gβ1 stabilize the β-propeller structure and reduce its contacts with CCT. These interactions facilitate release of Gβ1 from CCT to associate with Gγ subunits and form the stable Gβγ dimer [13].

The other PhLP proteins are less understood. PhLP2 is an essential gene in yeast and dictostelium [41,42], but it does not contribute to Gβ folding and Gβγ assembly [41,43]. Rather, yeast studies indicate that PhLP2 is a CCT co-chaperone in actin and tubulin folding [43,44]. They also suggest a co-chaperone role for PhLP2 in CCT-dependent folding of regulators of the G1 to S phase transition in the cell cycle [43]. A recent structure of a ternary complex between human actin, CCT, and PhLP2A (the human homolog of yeast PhLP2) provides insight into the co-chaperone role of PhLP2A. PhLP2A was identified as a density in the trans folding chamber with extended helices reaching through the CCT rings to contact the actin folding intermediate in the other chamber. The authors proposed that PhLP2A might act as an anchor to hold actin inside CCT upon ring opening to prevent premature release, which actin is prone to do, thereby slowing the rates of both actin release and ATP hydrolysis while simultaneously promoting proper actin folding [32]. The last member of the phosducin-like family, PhLP3, may also contribute to cytoskeletal function as a CCT co-chaperone, but in a different manner than PhLP2. Unlike PhLP2, genetic deletion of PhLP3 in yeast [41] and dictyostelium is not lethal [42], and PhLP3 overexpression does not rescue PhLP2 deletion [41]. Studies suggest that PhLP3 promotes β-tubulin folding [45–47], while it inhibits actin folding in yeast [45,46]. However, further work will be needed to clarify the CCT co-chaperone role of PhLP3.

Prefoldin is another well described CCT co-chaperone that is known to deliver nascent actin and tubulin to CCT for folding [48,49]. It consists of six homologous subunits that associate in a structure that resembles a jelly fish, with a body made up of a double β-barrel domain and six tentacles consisting of coiled-coils from each subunit. Prefoldin binds substrates via hydrophobic interactions within the tentacles and interacts with CCT to deposit substrates within the CCT folding chamber [50]. Recent *in vitro* work on actin folding has shown that prefoldin not only delivers actin to CCT, but also participates in the folding process to enhance CCT processivity and reduce actin aggregation [51]. Cryo-EM structures of prefoldin bound to CCT in the open form revealed two distinct conformations: a latched form in which prefoldin is bound but offset at an angle, and an engaged form in which the substrate binding chambers are aligned and the tentacles of prefoldin extend into the CCT folding chamber to modulate the substrate-binding surface [51]. Most notably, the engaged form recreates the asymmetric charge distribution observed on the walls of the closed CCT chamber. The authors propose that when actin binds incorrectly to CCT and cannot be properly folded, prefoldin will bind and fix its orientation to promote productive folding [51]. Collectively, these mechanistic descriptions reveal the important role of co-chaperones in increasing the diversity and efficiency of protein folding by CCT.

## CCT and disease

Given the large number of substrates folded by CCT, it comes as no surprise that CCT function is connected to a variety of diseases with a range of effects. For example, CCT exerts a protective effect against neurodegenerative diseases such as Huntingtin's and Parkinson's by inhibiting aggregation and promoting the clearance of aggregated proteins [17,52,53]. Loss of function mutations of CCT can cause Leber Congenital Amaurosis, a severe retinal degenerative disease [54], or recessive neuropathy [55]. CCT also folds the tumor suppressors p53 [56] and the von Hippel Lindau (VHL) protein [14,56]. In many cancers, however, CCT subunits are up-regulated and likely help to drive pro-survival signaling through the mTOR or STAT3 pathways among others [12,57–61]. Several CCT subunits were recently identified as potential biomarkers for head and neck squamous cancer because their up-regulation was associated with poor prognosis [62]. Additionally, anticarin-β, an inhibitor of CCT4, was shown to have a substantial anti-tumor effect on multiple models of osteosarcoma mediated through loss of STAT3 signaling [63].

Many viruses and other pathogens have evolved to co-opt CCT to fold their own proteins. Prominent examples include the gag, vif, and p6 proteins of HIV [64,65], the Zika virus Ns1 protein [66], and the reovirus σ3 capsid [67,68]. Cellular depletion of CCT severely impairs the replication of viruses that utilize it, indicating promise as an anti-viral therapeutic target [66,68–73]. Furthermore, the CCT-binding interface of viral proteins must be highly conserved as host chaperones do not evolve with the virus. Thus, antiviral drugs targeting these interfaces could avoid the common problem of rapid obsolescence as the virus mutates around them. Moreover, targeting CCT could produce broad-spectrum anti-viral drugs with a range of efficacy against groups of related viruses.

## Conclusion

The precise functions and mechanisms of CCT-mediated protein folding have long been a mystery. Previous models based on known mechanisms of other chaperonins do not satisfactorily account for its diverse substrate clientele or the range of other functions that have been observed. New structural studies, largely taking advantage of the striking improvements in cryo-EM capabilities in recent years, have finally started to fill in the gaps and demonstrate that the key to understanding CCT lies in its eight distinct subunits. Unique among chaperonins, the subunit arrangement of CCT provides broad diversity in binding sites, contact surfaces, and ATP hydrolysis rates. From this, we can construct a new model in which, rather than acting as a simple machine with a single rigid folding mechanism, CCT behaves much more like a complex, multifunction tool which can be utilized to accommodate the folding requirements of many different substrates. In this light, it becomes apparent that the evolution of CCT may well have been a critical factor in the rise of the eukaryotic proteome.

In terms of practical application, the observation that different CCT substrates have unique binding patterns and folding trajectories is significant. CCT is a challenging protein to inhibit because it is absolutely required for cell survival, yet it has potential as a therapeutic target because of its oncogenic effect in maintaining the proteome of cancer cells and its contribution to the replication of many virulent viruses. In the latter case especially, it could be a useful anti-viral target due to the stability of the interface between the chaperonin and viral proteins. Substrate-specific binding implies the possibility of substrate-specific inhibitors which could target one, or a small subset of substrate-CCT interactions without impairing the overall function of CCT. Continued structural studies of CCT folding with a broader range of substrates are needed to better explore this potential.

## Perspectives

- The CCT chaperonin participates in the folding of a wide variety of proteins and is indispensable for eukaryotic life, but also plays important roles in diseases ranging from cancer to viral infections.
- CCT actively directs substrate folding by providing specific binding sites and sequential release over multiple ATPase cycles. CCT accommodates an incredibly diverse set of substrates using a wide range of binding surfaces and potential folding trajectories offered by its eight distinct subunits.
- More structural studies of CCT in complex with different types of substrates and protein complexes are needed to complete the picture of CCT function. A detailed mechanistic understanding of its function will allow CCT to be targeted with therapeutic interventions.

## Abbreviations

CCT: Cytosolic chaperonin containing tailless complex polypeptide 1
Cryo-EM: Cryogenic electron microscopy
Gβ: G protein β subunit
Gγ: G protein γ subunit
HIV: Human immunodeficiency virus
Hsp10: Heat shock protein of 10 kDa
Hsp70: Heat shock protein of 70 kDa
Hsp90: Heat shock protein of 90 kDa
mLST8: Mammalian lethal with SEC13 protein 8 (subunit of mTOR complexes)
mTOR: Mammalian target of rapamycin
mTORC: Mammalian target of rapamycin complex
PhLP: Phosducin-like protein
RAPTOR: Regulatory associated protein of mTOR (subunit of the mTORC1 complex)
STAT: Signal transducer and activator of transcription
TRiC: Tailless complex polypeptide ring complex
WD-40: Sequence repeat of 40 amino acids often ending in tryptophan (W) and aspartate (D)
